# Supplementary material for: An open-source framework for large-scale, flexible evaluation of biomedical text mining systems
Source: J Biomed Discov Collab. 2008 Jan 29;3:1. doi: 10.1186/1747-5333-3-1 (PMC2276192; doi:10.1186/1747-5333-3-1)
Supplement: Additional file 1 — Evaluation platform source code. The additional file contains the software evaluation framework discussed in this paper. The most current release can be downloaded from SourceForge.net [1]. See the accompanying README file for instructions on installation and use. Also included in the distribution is a collection of UIMA wrappers for some commonly used BioNLP tools and annotated corpora. [file 1747-5333-3-1-S1.zip › bionlp_uima_repository-1.0.1/docs/api/edu/uchsc/ccp/util/nlp/annotation/printer/AnnotationToFileOutput.html]

AnnotationToFileOutput (Center for Computational Pharmacology Util/UIMA API)


|  |  |  |
| --- | --- | --- |
| **Overview** \| **Package** \| **Class** \| **Tree** \| **Deprecated** \| **Index** \| **Help** | |  |
| **PREV CLASS**   **NEXT CLASS** | **FRAMES**    **NO FRAMES**     **All Classes** |  |
| SUMMARY: NESTED \| FIELD \| CONSTR \| METHOD | DETAIL: FIELD \| CONSTR \| METHOD |  |


---


## edu.uchsc.ccp.util.nlp.annotation.printer Class AnnotationToFileOutput

```
java.lang.Object
  edu.uchsc.ccp.util.nlp.annotation.printer.AnnotationToFileOutput
```

**All Implemented Interfaces:**: AnnotationPrinterUtil

---

``` public class AnnotationToFileOutput extends Object implements AnnotationPrinterUtil ```

This Annotation Printer outputs annotation in a format that can be loaded by the AnnotationFromFileLoader. Currently, this will only work for
simple annotations. Use of the XCas is probably the best way to accomplish this task, but this will be used as we are only interested in storing
simple annotations (i.e. not slots) for the moment.  
  
The output consists of a single annotation per line taking the format:  
  
documentID|annotatorID|spanStart spanEnd|classMentionName|coveredText

---

**Constructor Summary**

`AnnotationToFileOutput()`


| **Method Summary** | |
| --- | --- |
| `void` | `printDocument(GenericDocument gd)` |
| `void` | `printDocument(GenericDocument gd, PrintStream ps)` |
| `void` | `printDocumentCollection(List<GenericDocument> documents)` |
| `void` | `printDocumentCollection(List<GenericDocument> documents, PrintStream ps)` |

**Methods inherited from class java.lang.Object**

`clone, equals, finalize, getClass, hashCode, notify, notifyAll, toString, wait, wait, wait`

**Constructor Detail**

### AnnotationToFileOutput

```
public AnnotationToFileOutput()
```


**Method Detail**

### printDocumentCollection

```
public void printDocumentCollection(List<GenericDocument> documents,
                                    PrintStream ps)
```

:   **Specified by:**: `printDocumentCollection` in interface `AnnotationPrinterUtil`

---


### printDocumentCollection

```
public void printDocumentCollection(List<GenericDocument> documents)
```

:   **Specified by:**: `printDocumentCollection` in interface `AnnotationPrinterUtil`

---


### printDocument

```
public void printDocument(GenericDocument gd)
```

:   **Specified by:**: `printDocument` in interface `AnnotationPrinterUtil`

---


### printDocument

```
public void printDocument(GenericDocument gd,
                          PrintStream ps)
```

:   **Specified by:**: `printDocument` in interface `AnnotationPrinterUtil`


---


|  |  |  |
| --- | --- | --- |
| **Overview** \| **Package** \| **Class** \| **Tree** \| **Deprecated** \| **Index** \| **Help** | |  |
| **PREV CLASS**   **NEXT CLASS** | **FRAMES**    **NO FRAMES**     **All Classes** |  |
| SUMMARY: NESTED \| FIELD \| CONSTR \| METHOD | DETAIL: FIELD \| CONSTR \| METHOD |  |


---
